# Learning Dictionaries of Sparse Codes of 3D Movements of Body Joints for Real-Time Human Activity Understanding

**DOI:** 10.1371/journal.pone.0114147

**Published:** 2014-12-04

**Authors:** Jin Qi, Zhiyong Yang

**Affiliations:** Brain and Behavior Discovery Institute, James and Jean Culver Vision Discovery Institute, Department of Ophthalmology, Georgia Regents University, Augusta, Georgia, 30912, United States of America; University of Verona, Italy

## Abstract

Real-time human activity recognition is essential for human-robot interactions for assisted healthy independent living. Most previous work in this area is performed on traditional two-dimensional (2D) videos and both global and local methods have been used. Since 2D videos are sensitive to changes of lighting condition, view angle, and scale, researchers begun to explore applications of 3D information in human activity understanding in recently years. Unfortunately, features that work well on 2D videos usually don't perform well on 3D videos and there is no consensus on what 3D features should be used. Here we propose a model of human activity recognition based on 3D movements of body joints. Our method has three steps, learning dictionaries of sparse codes of 3D movements of joints, sparse coding, and classification. In the first step, space-time volumes of 3D movements of body joints are obtained via dense sampling and independent component analysis is then performed to construct a dictionary of sparse codes for each activity. In the second step, the space-time volumes are projected to the dictionaries and a set of sparse histograms of the projection coefficients are constructed as feature representations of the activities. Finally, the sparse histograms are used as inputs to a support vector machine to recognize human activities. We tested this model on three databases of human activities and found that it outperforms the state-of-the-art algorithms. Thus, this model can be used for real-time human activity recognition in many applications.

## Introduction

**Figure 1:**
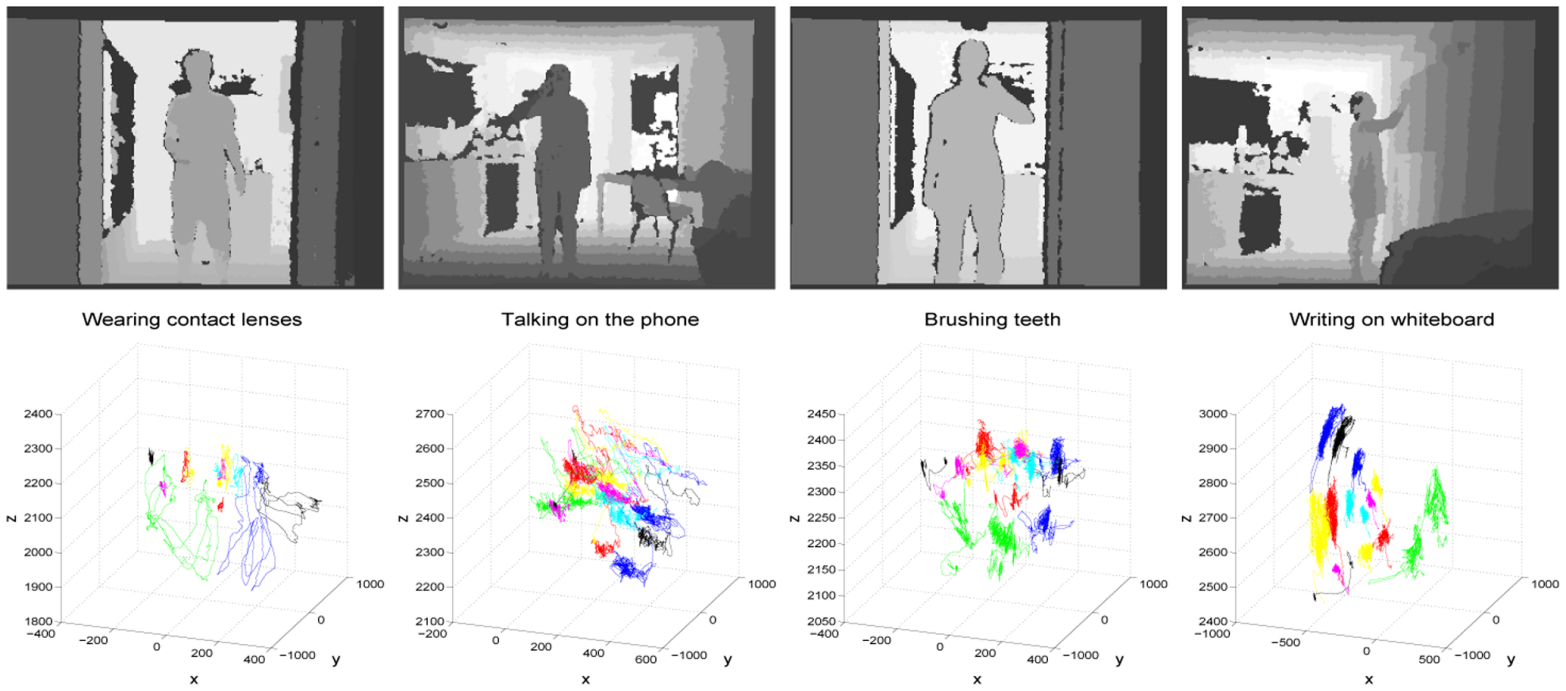
Four activities in the CAD-60 dataset. First row: depth images; Second row: joint trajectories.

A smart environment is a place where humans and objects (including mobile robots) can interact and communicate with each other in a human-like way [1]. It has a wide range of applications in home and office work, health care, assistive living, and industrial operations. Current pervasive computing technologies and low-cost digital imaging devices make feasible the development of smart environments. In smart environments, accurate, real-time human activity recognition is a paramount requirement since it allows to monitor individuals/patient's activities of daily living [2], such as taking medicine, dressing, cooking, eating, drinking, falling down, and feeling painful, to keep track of their functional health, and to timely intervene to improve their health [3]–[7]. Fig. 1 shows several human activities in the dataset CAD-60 [8], including “wearing contact lens”,“talking on the phone”, “brushing teeth” and “writing on the white board”.

Automated human activity understanding is a challenging problem due to the diversity and complexity of human behaviors [9]. Different people do the same activity in a multitude of ways; and even for a single person, he or she may do the same activity in different ways at different times. Most previous work in human activity understanding is performed on traditional 2D color images/videos and both global and local spatial-tempo features have been proposed (reviewed in [10]–[12]). Because it is difficult to deal with variations in 2D images/videos due to changes in lighting condition, view angle, and scale, researcher begun to explore applications of 3D information in human activity understanding [9]. In contrast to 2D images/videos, depth maps such as those acquired by the Microsoft Kinect system are related to object geometry and thus are independent of lighting conditions.

However, it is a difficult task to develop features to representation human activities based on 3D information. This is because depth images have much less textures than 2D images and are sensitive to occlusion [13]. Adopting recognition algorithms developed to work on 2D images and videos is not trivial either. For example, interest-point detectors such as Dollar [14] and STIP [15] perform badly on 3D videos. Currently, there are two approaches in using depth data for activity recognition, depth based and skeleton/joint based methods [9]. A recent study showed that relative joint positions carry significant information about activities [16], but these features are difficult to extract without human intervention. Thus, although several recognition algorithms that use manually selected joint-related features have been developed [8], [17]–[24], there is no consensus on what joint-related features should be extracted and how they should be used for activity recognition.

We propose a method that learns automatically sparse representations of human activities. Specifically, we treat 3D movements of joints as space-time volumes and densely sample the volumes along the time axis to obtain a set of sub-volumes. We then use the reconstructed independent component analysis (RICA) [25] to learn a dictionary of over-complete codes from the sub-volumes for each activity. In this learning procedure, the sub-volumes are represented by the learned codes in a sparse manner. From the coefficients of the sub-volumes projected to the sparse codes, we construct a sparse histogram for each activity. Finally, we concatenate the sparse histograms and use them as inputs to a multi-class support vector machine (SVM) to perform activity recognition.

We tested this model on three widely used databases of human activities and found that it outperforms the state-of-the-art algorithms. The contributions of this paper to joint-based activity recognition are:

- a general dictionary-based framework that automatically learns sparse, high-dimensional spatial-temporal features of 3D movements of joints,
- an efficient method that constructs sparse codes and histograms,
- a real-time system for human activity recognition that can be easily implemented,
- extensive evaluations on the proposed model and superior results on three datasets of human activities.

The paper is organized as follows. In Section 2, we briefly describe related work and how our model is different. In Section 3, we describe the procedures of data processing and learning dictionaries of codes of 3D movements of body joints. In Section 4, we propose a set of sparse histograms of the codes of human activities. In Section 5, we present an algorithm for activity recognition via a multi-class SVM with sparse histograms as input features. In Section 6, we report the recognition results of our model on three datasets of human activities and compare them to the state-of-the-art algorithms. In Section 7, we briefly summarize the main points of our model and address several aspects of the model that can be improved.

## Methods

### 2.1 Related Work

We briefly describe related work below. For work on activity recognition based on 2D videos, we refer readers to several surveys [10]–[12].

#### Depth map-based approaches

Features automatically or manually extracted from depth images/videos have been proposed, including bag of points [26], Space-Time Occupancy Patterns (STOP) [27], Random Occupancy Pattern (ROP) [28], HOG from Depth Motion Maps (DMM-HOG) [24], Histogram of Oriented 4D Surface Normals (HON4D) [29], Pixel Response and Gradient Based Local Feature [30], Local Trajectory Gradients, and SIFT [31]. In [32], depth silhouettes are used as features and a hidden Markov Model (HMM) is used to model temporal dynamics of activities. Different from these methods, our algorithm is based on joints which are the best features for human activity recognition [16].

#### Skeleton/Joint based approaches

It was observed in 1970's that a range of human activities can be recognized on the basis of 3D movements of body joints [33]. However, joint-based activity recognition drew research attention only recently due to the availability of low-cost Microsoft Kinect cameras that can acquire 3D videos of joint movements. Campbell and Bobick [17] proposed to compute action curves by projecting 3D joint trajectories on low-dimensional phase spaces and to classify actions based on action curves. This approach works only for simple activities. Lv et al. [18] proposed seven types of local features and used HMMs to describe the evolution of these features. In [19] a so-called Histogram of 3D Joint Location (HOJ3D) was designed to characterize the distribution of joints around the central joint (hip joint) and a HMM was developed to model temporal changes of the feature. In [20], SIFT features for objects and skeleton features for humans were developed and an MRF was used to model human activities. Sung et al. [8] computed HOG from RGBD data and position-angle features from joints and used a Maximum Entropy Markov Model (MEMM) to represent activities hierarchically. Wang et al. [34] designed Local Occupancy Pattern (LOP) which was computed from a set of 3D points around each joint. Finally, geometric relationships among joints were used in [23]. All these methods need manually designed features. In contrast, a set of dictionaries of sparse codes of human activities are obtained without manual interventions in the method we present here.

The work related to ours is the EigenJoints that describe positional differences between joints within or cross video frames and are used for action recognition via a Naive Bayes nearest neighbor classifier [24]. The EigenJoints are simple and easy to compute and so are the features of our model presented below. Our model is different in two ways. First, a set of dictionaries of codes of human activities are learned. Second, an approximate sparse coding is performed to obtain a set of sparse histograms for action recognition via a multi-class SVM.

### 2.2 Joint-Dictionary Learning

We propose to learn a set of dictionaries of sparse codes to represent the complex spatial-temporal relationships among body joints. For this purpose, we introduce some notations first.

#### 2.2.1 Notations

The *d*-th video is denoted by 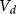 and the total number of frames in the *d*-th video 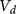 is 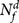. The number of joints in each frame 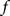 is denoted by 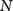 and the 3-dimensional coordinate vector of each joint 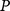 in frame 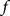 is 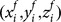.

#### 2.2.2 Sampling space-time-joint volume

**Figure 2:**
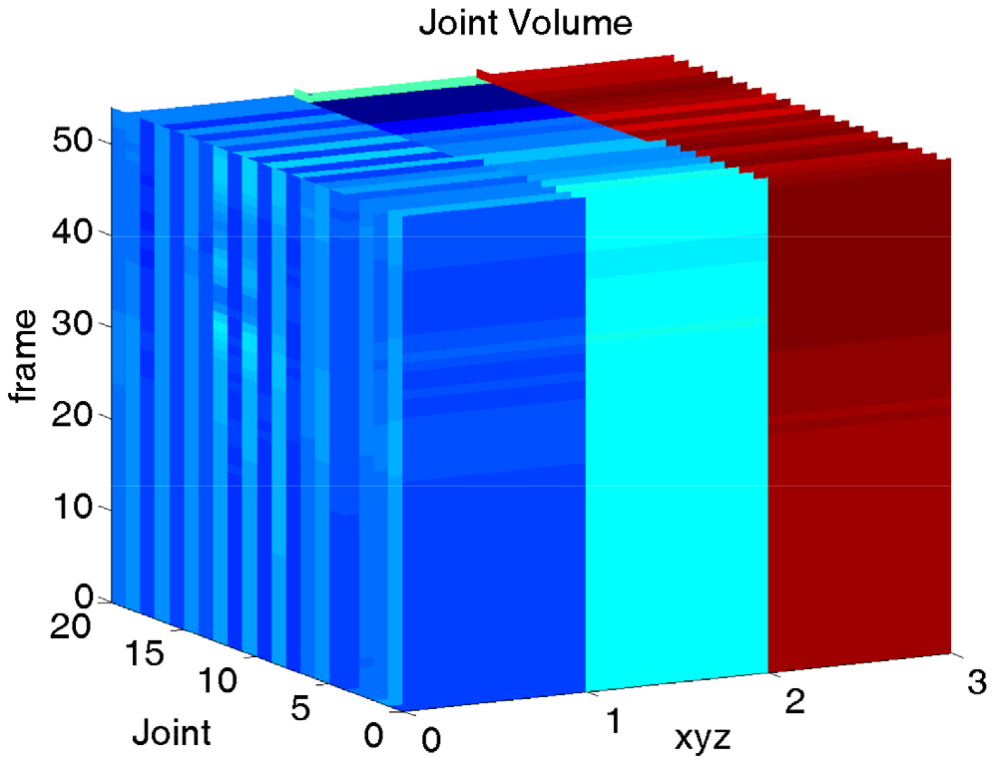
Joint volume. xyz axis: joint coordinates (x,y,z); joint axis: indices of joints; frame axis: index of video frames.

For each frame 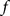, we construct a matrix 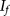 by concatenating all the coordinates of 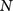 joints in frame 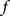. Specifically, the 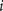-th row of 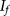 is the coordinate vector 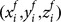 of the 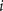 th joint in frame 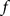 and the columns of 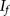 are the 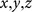 coordinates of the joints. Therefore, the size of 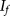 is 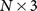. Each column 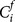 of the matrix 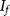 is subtracted by its mean 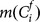. This operation makes 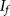 invariant to camera/human placements. Although this operation removes global body motion, it won't affect much the performance of the model developed here since the activities in the three tested datasets are indoor human daily activities that don't entail much global body motion. We then concatenate all the matrices 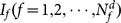 from the *d*-th video 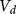 to form a volume 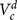 as shown in Fig. 2. 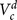 is a matrix of a dimension of 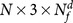. Mathematically, we have
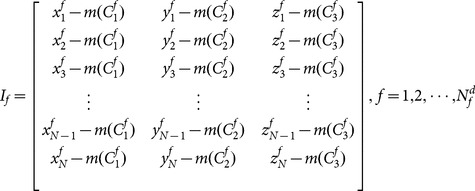
(1)and 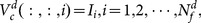
(2)where 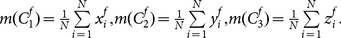
(3)


We densely sample 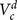 along the time dimension (“frame” axis in Fig. 2) to obtain 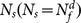 sub-volumes for each video. Thus, we take all possible sub-volumes of 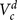. One can use various methods to take sub-volumes at sampled points in the time dimension (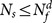). Suppose that the sample sizes are 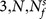 along the “xyz“ axis, the “joint” axis, and the “frame” axis, respectively. Each sample 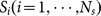 is then a 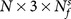 sub-volume sampled from 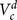, which can be written as
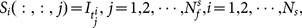
(4)where 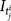 is the 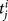
th coordinate image.

**Figure 3:**
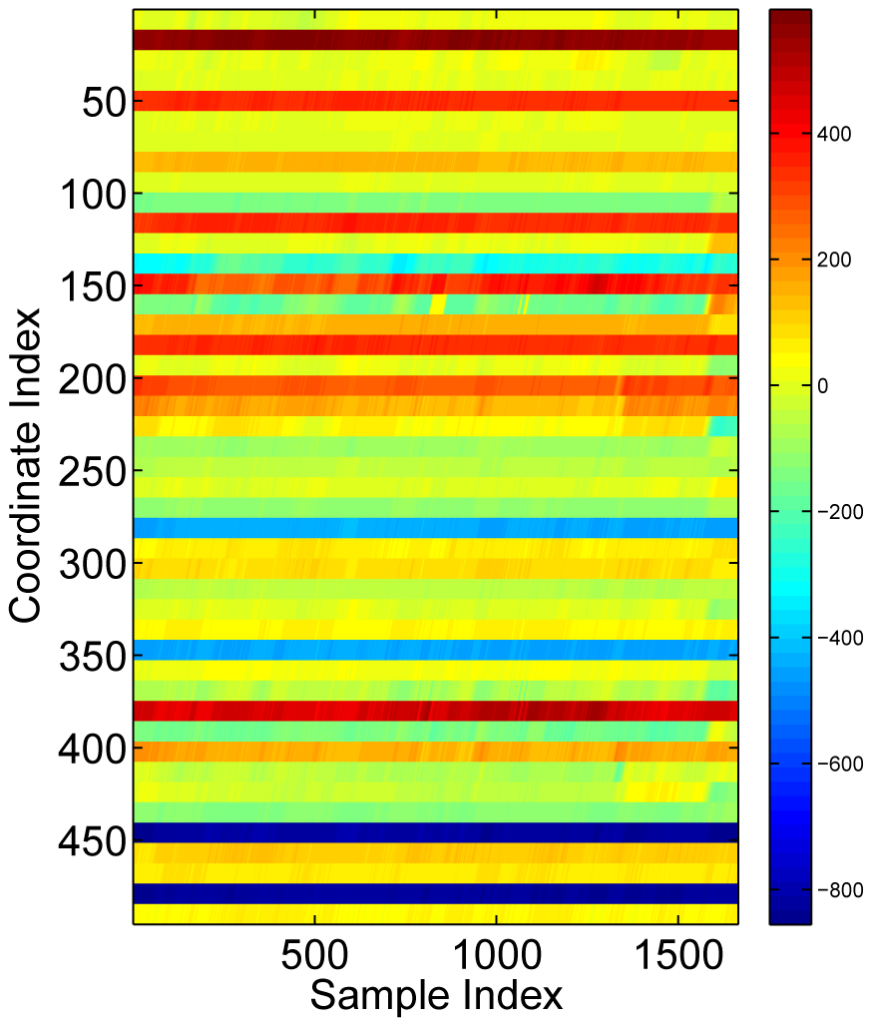
Coordinate samples from one video. Each column corresponds to one coordinate sub-volume sample. The “Sample index” axis indicates the indices of all sub-volume samples and the “Coordinate index” axis is the row index of matrix 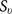.


The third dimension of sub-volume 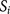 can be permuted with the first dimension by a permutation operation 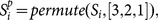
(5)where vector 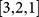 indicates that the second dimension of 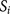 stays where it is but the first dimension is swapped with the third dimension. From equation (1), it can be seen that the same coordinate components of each joint form the columns of the permuted sub-volume 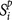 by the above permutation operation. As a result, either 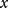, 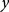, or 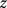 coordinate components of a joint in the sampled frames in sub-volume 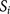 form one column of the permuted sub-volume 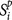. For example, the 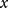 coordinate components of the head joint in different frames in sub-volume 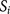 are one column of 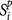. This is illustrated by the horizontal color bars in Fig. 3 since body joints in neighboring frames tend to have similar coordinates. To examine the sub-volumes, we form a new matrix 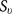 by by reordering the permuted sub-volumes 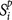 lexicographically. 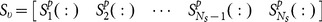
(6)


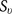
 represents the sub-volumes from one video with each column corresponding to one sub-volume. One 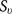 is shown in Fig. 3, where the “Sample index” axis indicates the indices of all the sub-volume samples and the “Coordinate index” axis is the row index of matrix 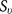. As shown in Fig. 3, gradual changes between samples occur along the “Sample index” axis (corresponding to time axis). Thus, the configurational relationships among body joints update in the time domain, as they should in human activities.

#### 2.2.3 Semantics of space-time-joint sub-volumes

The *i*-th sub-volume 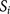 described above contains several video frames (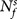 frames) which may capture components of one or more activities. For big 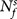, there are more frames in a sub-volume, which may capture an activity. For small 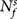, there are few frames in a sub-volume, which may only capture a part of an activity. Two extreme cases are 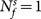 and 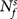 is equal to the total number of the frames of the videos.

In following section, we propose to learn a set of dictionaries of codes that can be used to represent complex human activities. The words (i.e., codes) in the dictionaries should be components whose concatenations in the space and time domains constitute representations of human activities. Thus, 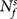 should be neither too small nor too big so that the sub-volumes are samples of components of human activities. Unfortunately, it is difficult to set a fixed value for 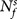 for all human activities, which may have components of a variety of spatial and temporal scales and may be captured by cameras of a range of imaging parameters. Therefore, we set the values of 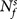 via a learning procedure for the three datasets tested in this paper.

#### 2.2.4 Joint-dictionary learning

We propose a method to learn a set of sparse codes that can be used to represent human activities. Sparse representation is useful for object recognition [25]. A number of algorithms have been proposed to learn sparse features, including restrict Boltzmann machines [35], spare auto-encoder [36], independent component analysis [37], sparse coding [38], and RICA [25]. Since RICA works well on approximately whitened data and is fast [25], we use RICA to learn a dictionary of codes from a set of sub-volumes 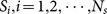 for each activity. The learned dictionary is called “Joint-dictionary”. To the best of our knowledge, this is the first work on feature learning from 3D movements of body joints.

For each activity 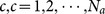 (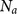 is the number of activities), we obtain a dictionary 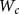. Suppose 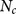 is the total number of sub-volume samples from activity 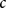. Then the class-specific dictionary 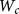 can be obtained by solving the following optimization problem [25]

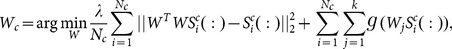
(7)where 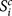 is the *i*th sub-volume sample from activity 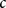; 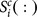 is a lexicographical operation on 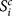 to form a column vector; 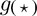 is a nonlinear convex function (e.g., smooth 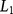 penalty function 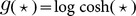 [39] in this paper); and 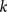, 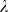 are the number of features (rows of 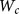) and a balancing parameter, respectively.

The objective function in (7) is a smooth function. The optimization problem (7) can be easily solved by any unconstrained solvers (e.g., L-BFGS and CG [40]).

We propose to learn a class-specific dictionary 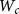 for each activity 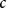 and we pool all the learned class-specific dictionaries 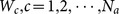 to form a code book 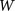 as follows 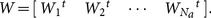
(8)


The code book 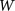 contains 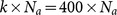 words in total. Note that 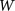 is over-complete since the number of words is bigger than the size of sub-volumes.

**Figure 4:**
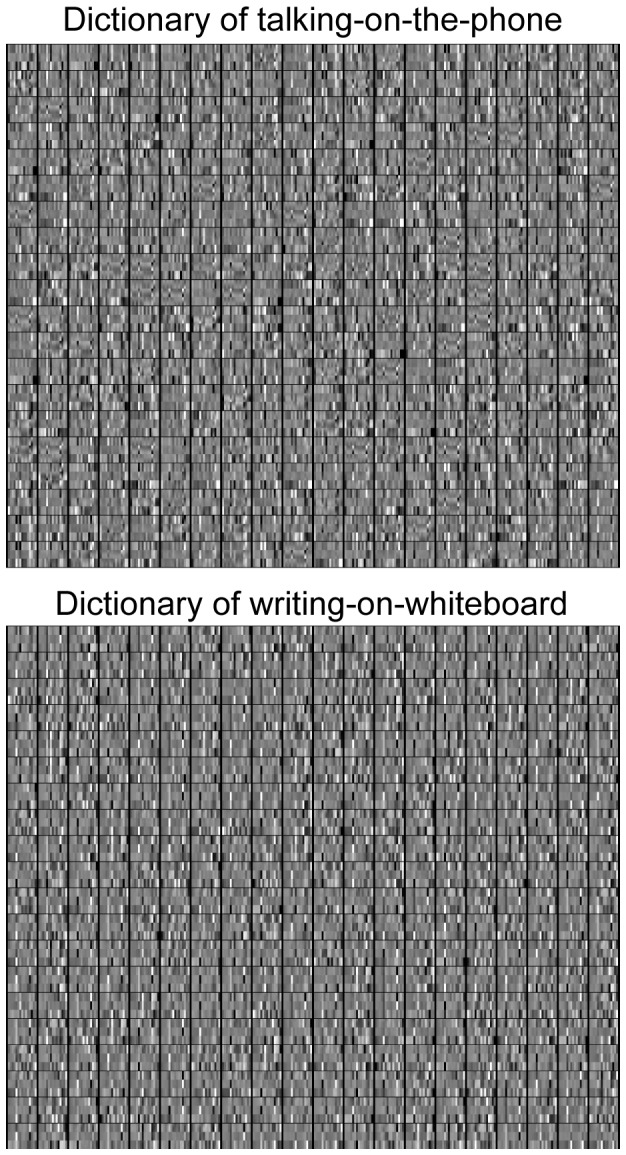
Two dictionaries for “talking on the phone” (top) and “writing on white board” (bottom). 400 words are shown in 400 squares.

Fig. 4 shows two dictionaries for “talking on the phone” and “writing on white board”. Each dictionary contains 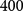 words. The words shown in Fig. 4 are used to represent 3D spatial-temporal sub-volumes and are different from conventional words (e.g., oriented bars) learned from 2D natural image patches [25]. These words are the bases of segments of space-time concatenations of body joints by which any segment of an activity can be constructed linearly. Unfortunately, unlike independent components of natural scenes, which are like edge elements, the words obtained here are difficult to visualize.

### 2.3 Sparse Histograms

In this section, we propose an approximate sparse coding scheme and compile a set of sparse histograms. Any sample 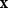 can be sparsely represented by 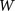 as following 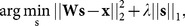
(9)where 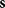 is the sparse coefficients of sample 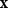 represented by dictionary 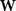. A number of algorithms have been proposed to solve the above problem of sparse representation [41].

Instead of solving the optimization problem (9) for each video, which is prohibitively time consuming, we propose to project any sample 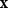 onto 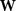 via 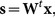
(10)where 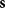 is the coefficients of sample 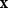. The first 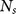 (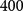 in this paper) largest coefficients are kept and the rest coefficients of 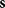 are set to zero to make 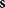 sparse. Note that the dimension of 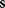 is 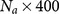 (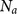 is the number of activities). The number of the kept sparse coefficients (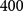 in this paper) seems to be big, but it is a lot smaller than the dimensionality of sub-volumes, which is 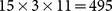 for the CAD60 database, and the dimensionality of the entire video. In the Section 6 we show that 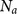 can be much smaller while good performance on activity recognition can be still achieved by our method.

The computation in equation (10) is very fast. Although this is an approximate sparse coding scheme, our results show that this approximation does not impair activity recognition (see Section 6).

**Figure 5:**
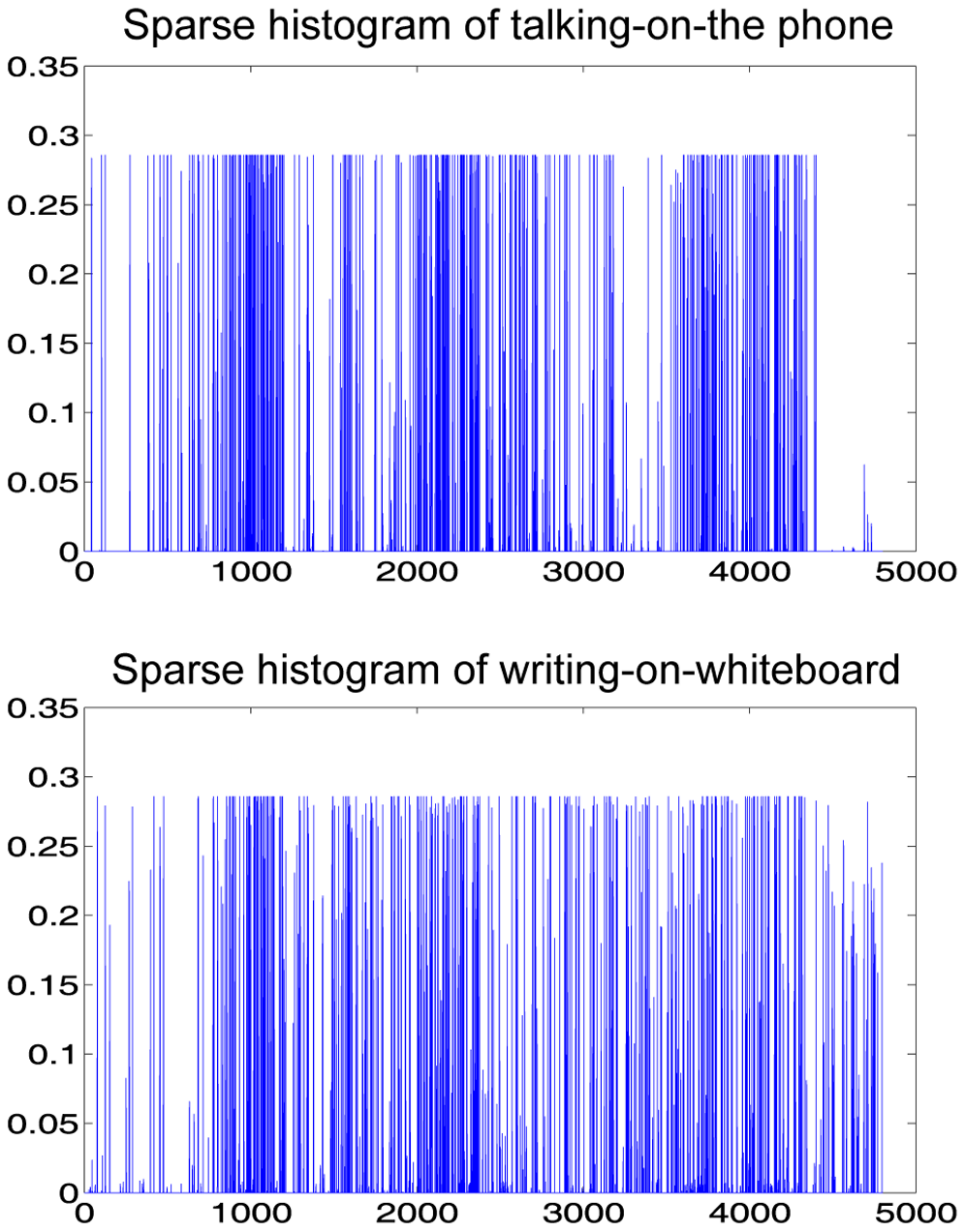
Sparse histograms of “talking on the phone” and “writing on the white board” in the CAD-60 dataset. The sparsity degrees are 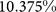 and 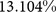 respectively. Note that the sum of the histograms is 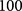.



We then obtain the histogram 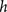 of nonzero coefficients of samples of a video 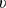 by counting the number of occurrences of nonzero coefficients for each word in 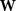. Thus, the *i*th component of 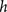 is the number of occurrences of the *i*th word that appears in video 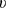. Fig. 5 shows the histograms of “talking on the phone” and “writing on the white board” of the CAD-60 database. The two histograms are quite different upon a careful visual examination. We define the degree of sparsity of a histogram as the ratio of the number of non-zero bins to the bin size

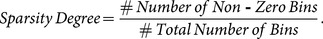
(11)


The sparsity degrees of the two histograms in Fig. 5 are 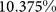 and 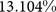, respectively. Thus, the histograms constructed this way are sparse.

Note that the histogram bins in Fig. 5 have more or less the same height (about 0.3). This may be due to similar words in the dictionaries for the activities in the dataset. Since a dictionary is learned from each activity independently, it is likely that there are words that are shared by more than one activities. It is worthy to point out, though, that shared words do not impair the performance of our algorithm.

### 2.4 Classification

**Figure 6:**
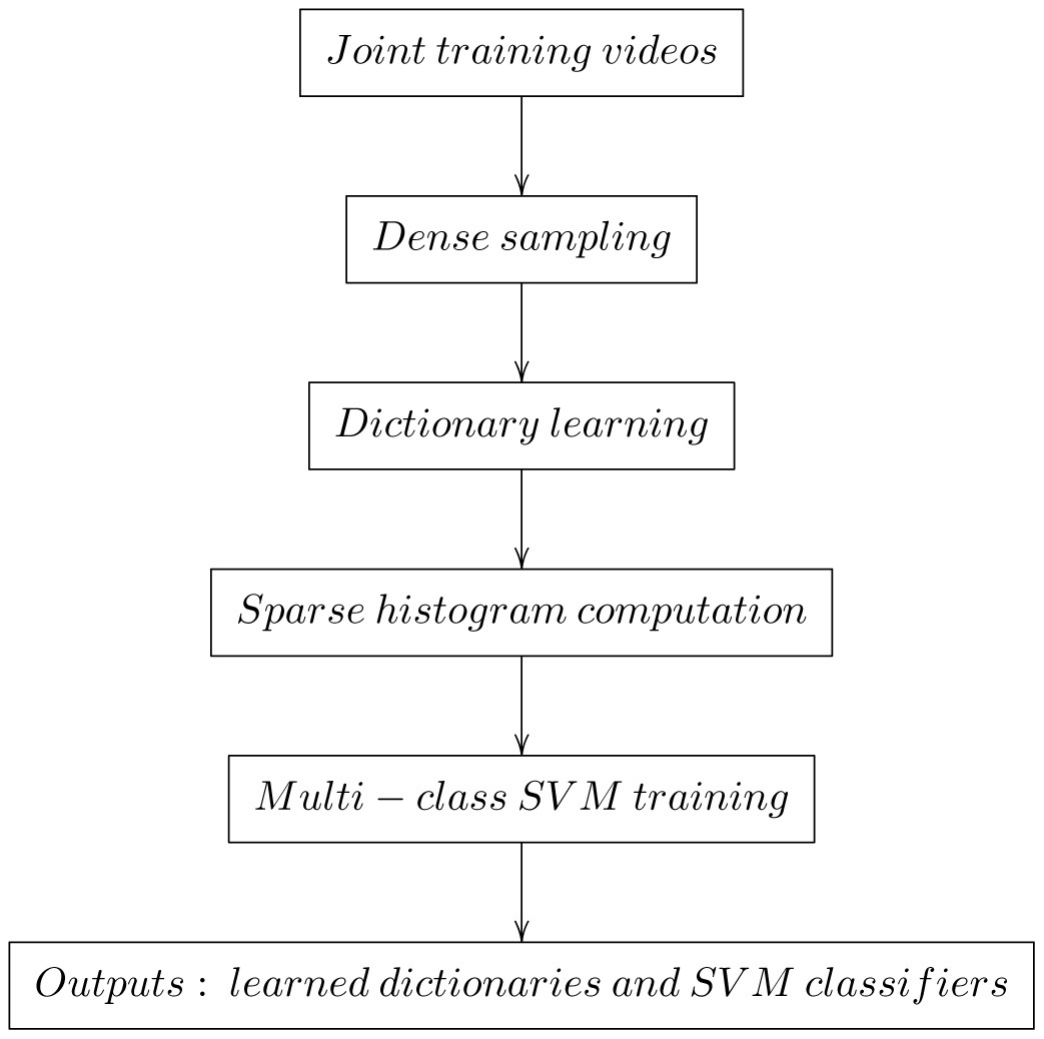
Flow chart for learning dictionaries and SVM classifiers.

**Figure 7:**
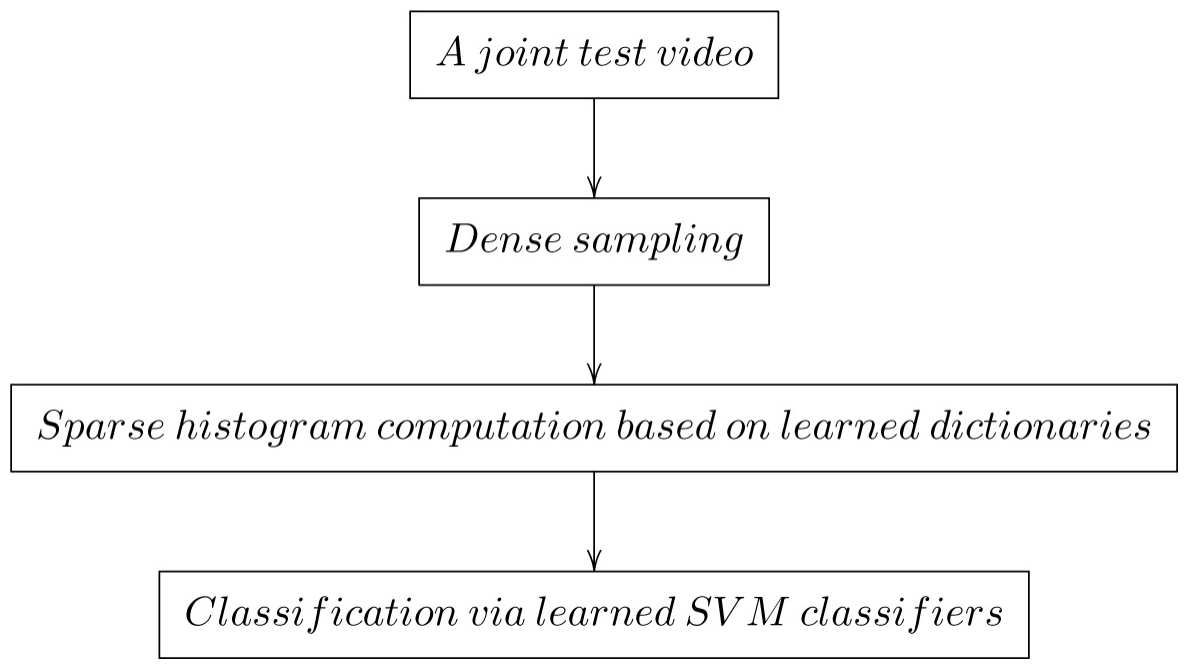
Flow chart for human activity recognition.

We compile a sparse histogram for each activity and use it as a feature for recognition via a multi-class SVM. In this procedure, we train one SVM in a one-vs.-rest scheme for each activity; use the homogeneous kernel map expansion [42] with a “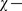 square” kernel to expand the dimensionality of feature by 2 times; and implement the computing with the source codes of an open-source collection of vision algorithms called “VLFeat” (http://www.vlfeat.org/). The training and testing procedures are summarized in Fig. 6 and Fig. 7, respectively.

## Results

We tested our algorithm on three publicly available datasets: the Cornell Activity Dataset-60 (CAD-60) [8], the MSR Action3D [43], and the MSR Daily Activity 3D [22]. Our results show that the model proposed here is better than the state-of-the-art methods.

### 3.1 CAD-60 dataset

The CAD-60 dataset is an RGBD dataset acquired with a Microsoft Kinect sensor at 30 Hz and has a resolution of 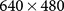 pixels [8] (Dataset S1). The 3D coordinates of 15 joints are the real-time outputs of the skeleton tracking algorithm of the sensor [44]. The dataset contains 14 human activities performed indoors by 4 subjects (two males and two females) for about 45 seconds. The total number of frames of each activity of each person is about one thousand. We follow the “new person” setting in [8] where data of 3 subjects were used for training and the remaining one subject for testing. To improve recognition performance, we mirrored the joints of the left-handed subject to make her activities similar to those of the other 3 right-handed subjects, which is a usually practice. Briefly, a plane 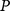 was first found by fitting four joints, left-arm, right-arm, left hip, and right hip. Then, a mirror plane 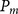 was computed under the constraints that 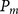 is perpendicular to 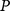 and passes through the middle point between the two arm joints and through the middle point between the two hip joints. Finally, all joints of the left-handed subject were mirrored with respect to 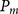.

**Figure 8:**
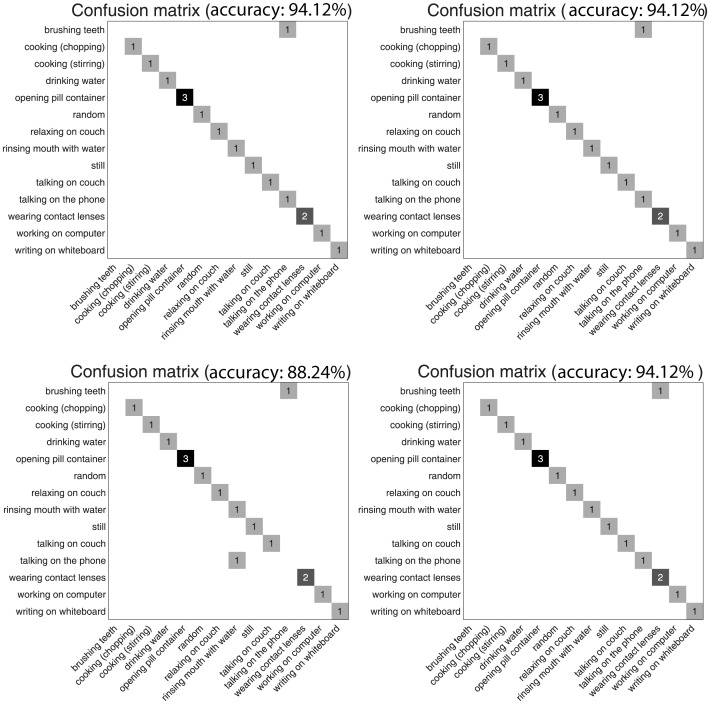
Four confusion matrices for four experimental settings.

Fig. 8 shows 4 confusion matrices for four cases where three subjects are chosen for training and the remaining subject for training. We compare our results to 9 algorithms in terms of average accuracy, precision, and recall in Table 1. The results of other algorithms are from the website http://pr.cs.cornell.edu/humanactivities/results.php that reports results on the dataset. As shown in Table 1, our algorithm is the best in terms of accuracy, precision, and recall on this dataset. Since some authors reported the performance of their algorithms in terms of only part of the above metrics, there are blank cells in Table 1.

**Table 1:** Performance of our model and other methods on the CAD-60 dataset.

Algorithm	Accuracy (%)	Precision (%)	Recall(%)
Sung et al., AAAI PAIR 2011, ICRA 2012 [8].		67.9	55.5
Koppula, Gupta, Saxena, IJRR 2012 [20]		80.8	71.4
Zhang, Tian, NWPJ 2012 [46]		86	84
Ni, Moulin, Yan, ECCV 2012 [47]	Accur: 65.32		
Yang, Tian, JVCIR 2013 [48]		71.9	66.6
Piyathilaka, Kodagoda, ICIEA 2013 [49]		70*	78*
Ni et al., Cybernetics 2013 [50]		75.9	69.5
Gupta, Chia, Rajan, MM 2013 [51]		78.1	75.4
Wang et al., PAMI 2013 [52]	Accur: 74.70		
**Ours**	**91.17**	**89.11**	**89.28**

### 3.2 MSR Action3D dataset

The MSR Action3D dataset contains 20 activities acquired from 10 subjects, each of whom performed each activity 2 or 3 times. The resolution is 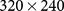 pixels and the frame rate is 16 Hz. The dataset provides the 3D movement data of 20 joints per person. We used 557 videos out of the 567 videos in the dataset since 10 videos have missing joints or erroneous joints [22] (Dataset S2).

To allow fair comparison, we followed the same setting as [22]: subjects Nos. 1, 3, 5, 7, and 9 as the training set and subjects Nos. 2, 4, 6, 8, and 10 as the testing set. The 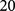 actions are divided into three subsets, AS1, AS2, and AS3 according to the experimental setting in [22], [43], which are listed in Table 2. AS1 and AS2 contain similar actions and AS3 contains complex actions composed of simpler ones.

**Table 2:** Subsets of actions, AS1, AS2, and AS3 in the MSR Action 3D dataset.

Action Set 1(AS1)	Action Set 2 (AS2)	Action Set 3(AS3)
Horizontal arm wave	High arm wave	High throw
Hammer	Hand catch	Forward kick
Forward punch	Draw x	Side kick
High throw	Draw tick	Jogging
Hand clap	Draw circle	Tennis swing
Bend Two	hand wave	Tennis serve
Tennis serve	Forward kick	Golf swing
Pickup & throw	Side boxing	Pickup & throw

**Figure 9:**
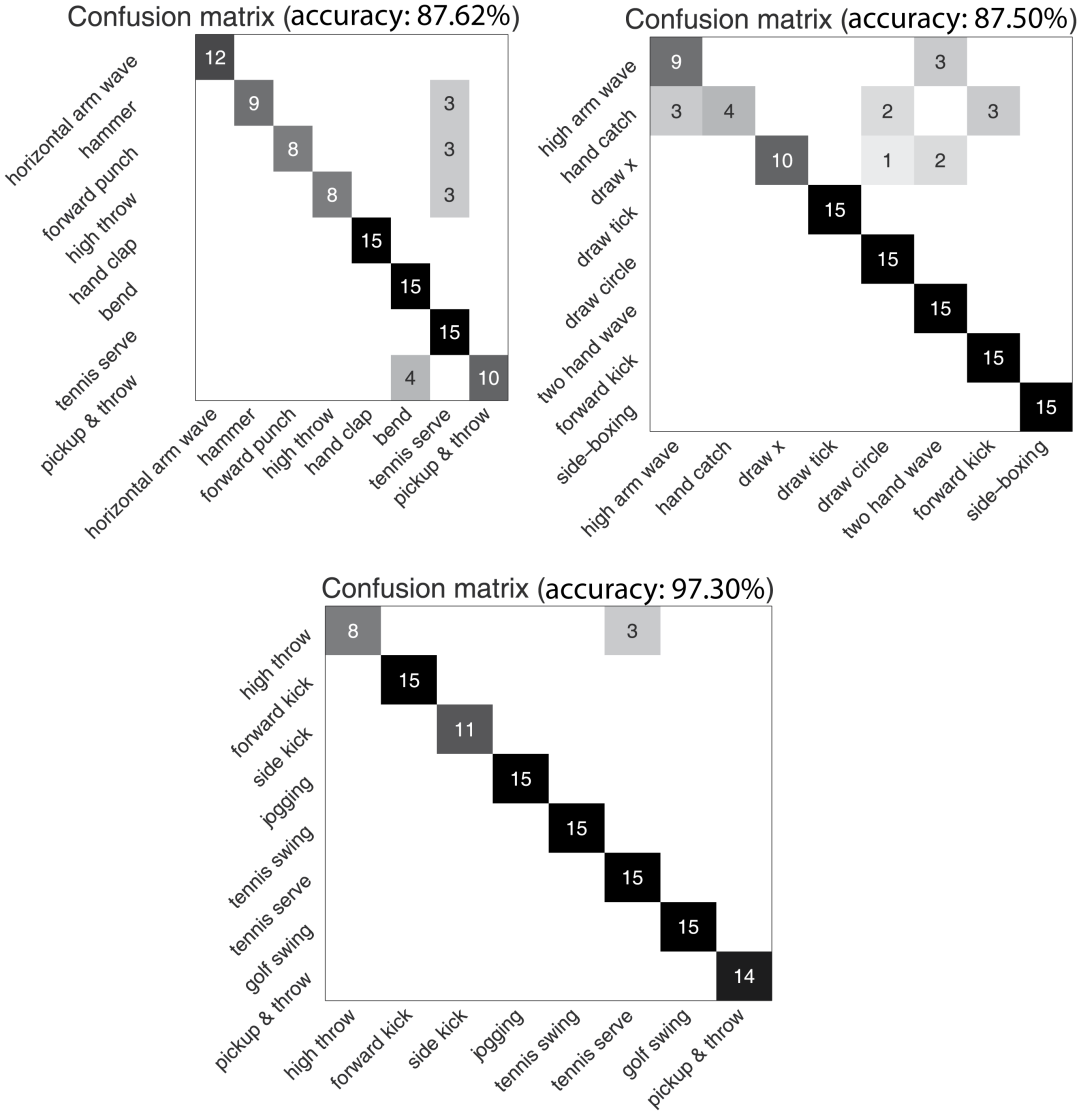
Three confusion matrices for AS1, AS2, AS3 in the MSR Action 3D dataset.

The accuracy of our algorithm on AS1, AS2 and AS3 is 87.62%, 87.5% and 97.3%, respectively. The average accuracy on the dataset is 90.81%. The three confusion matrices for AS1, AS2, and AS3 are shown in Fig. 9. Thus, our algorithm performs better on AS3 than AS1 and AS2.

Table 3 compares the performance of our model to other 9 methods. The accuracies of methods are from a recent paper [29]. The performance (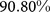) of our model is the best.

**Table 3:** Performance of our model and other methods on the MSR Action 3D dataset.

Method	Accuracy (%)
HON4D + Ddiscb [29]	88.89
HON4D [29]	85.85
Jiang et al. [22]	88.20
Jiang et al. [34]	86.50
Yang et al. [24]	85.52
Dollar + BOW[14]	72.40
STIP + BOW [15]	69.57
Vieira et al. [27]	78.20
Klaser et al. [53]	81.43
**Ours**	**90.81**

### 3.3 MSR Daily Activity 3D dataset

The MSR Daily Activity 3D dataset contains 16 activities each of which was performed twice by 10 subjects [22] (Dataset S3). The dataset contains 320 videos in each of 3 channels, RGB, depth, and joint. There are 20 body joints recorded whose positions are quite noisy due to two poses: “sitting on sofa” and “standing close to sofa”.

**Figure 10:**
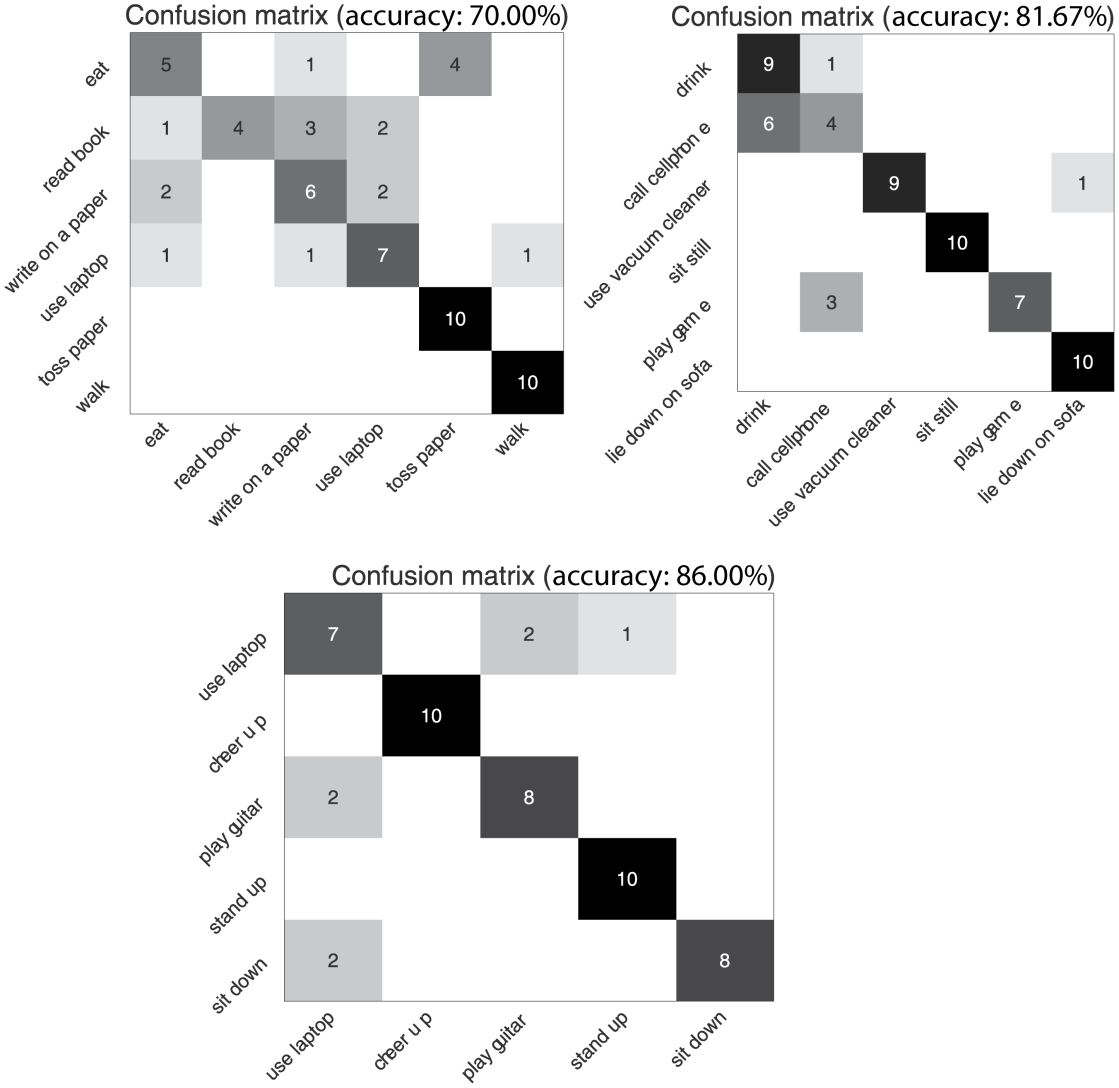
Three confusion matrices for AS1, AS2, AS3 in the MSR Daily Activity 3D dataset.

The experimental setting is the same as in [22] which split the dataset into 3 subsets, AS1, AS2, and AS3 as listed in Table 4. We followed the same setting as [22]: subjects Nos. 1,3,5,7, and 9 as the training set and subjects Nos. 2,4,6,8,and 10 as the testing set. The accuracy of our algorithm on AS1, AS2 and AS3 is 71.67%, 81.25%, and 85.00%, respectively and the average accuracy is 79.31%. The confusion matrices are shown in Fig. 10. Our algorithm performs better on AS3 than AS1 and AS2.

**Table 4:** Subsets of actions, AS1, AS2, and AS3 in the MSRDaily Activity 3D dataset.

Action Set 1(AS1)	Action Set 2 (AS2)	Action Set 3(AS3)
eat	drink	use laptop
read book	call cellphone	cheer up
write on a paper	use vacuum cleaner	play guitar
use laptop	sit still	stand up
toss paper	play game	sit down
walk	lie down on sofa	

Table 5 lists the results of our model and several other methods. The results of other methods are from a recent paper [22]. The accuracy of our model is 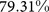 which is lower than the best result (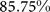). However, only joint information is used in our model while both joint and depth information is used to obtain the best result [22]. Compared to other models that use only joint information, our model is the best, outperforming the best earlier result which is 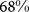.

**Table 5:** Performance of our model and other methods on the MSR Daily Activity 3D dataset.

Method	Accuracy(%)
Dynamic Temporal Warping [54]	54
Only LOP features[22]	42.5
Only Joint Position features [22]	68
SVM on Fourier Temporal Pyramid Features [22]	78
Actionlet Ensemble [22]	85.75
**Ours**	**79**

### 3.4 Comparison with a baseline method

We have evaluated the performance of our method on three public datasets. Our method has four steps: generating samples, learning dictionaries, constructing sparse histograms, and classifying via SVMs. In this section, we replace the RICA-based dictionary learning in our method with the k-means clustering. We cluster samples with the k-means algorithm and take the clusters as words in the dictionaries. We call this method as a baseline method. The results of this baseline method and our original method on the three datasets are shown in Table 6. Both methods perform well, with our original method being slightly better. Thus, the joint dictionaries and sparse histograms in both methods are responsible for the good performance.

**Table 6:** Performance of our model and the baseline method on the three databases.

	CAD-60	MSRAction3D	MSRDaily Activity3D
Our method	91.17	90.81	79
Baseline method	89.71	88.34	77.03

### 3.5 Parameter setting and time performance

There are seven parameters in our model. They are 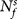, the sampling size along the z-direction; 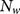, the number of words in each class-specific dictionary; 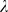, the balancing parameter in Eq. 7; 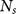, the number of the largest coefficients; 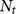, the factor by which the dimensionality of feature vector is expanded; 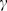, the parameter of the 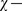 square kernel; and 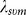, the balancing parameter of the SVM. These parameters are probably independent of each other since they are for different phrases of our algorithm, sampling, dictionary learning, sparse histogram, and SVM training.

**Figure 11:**
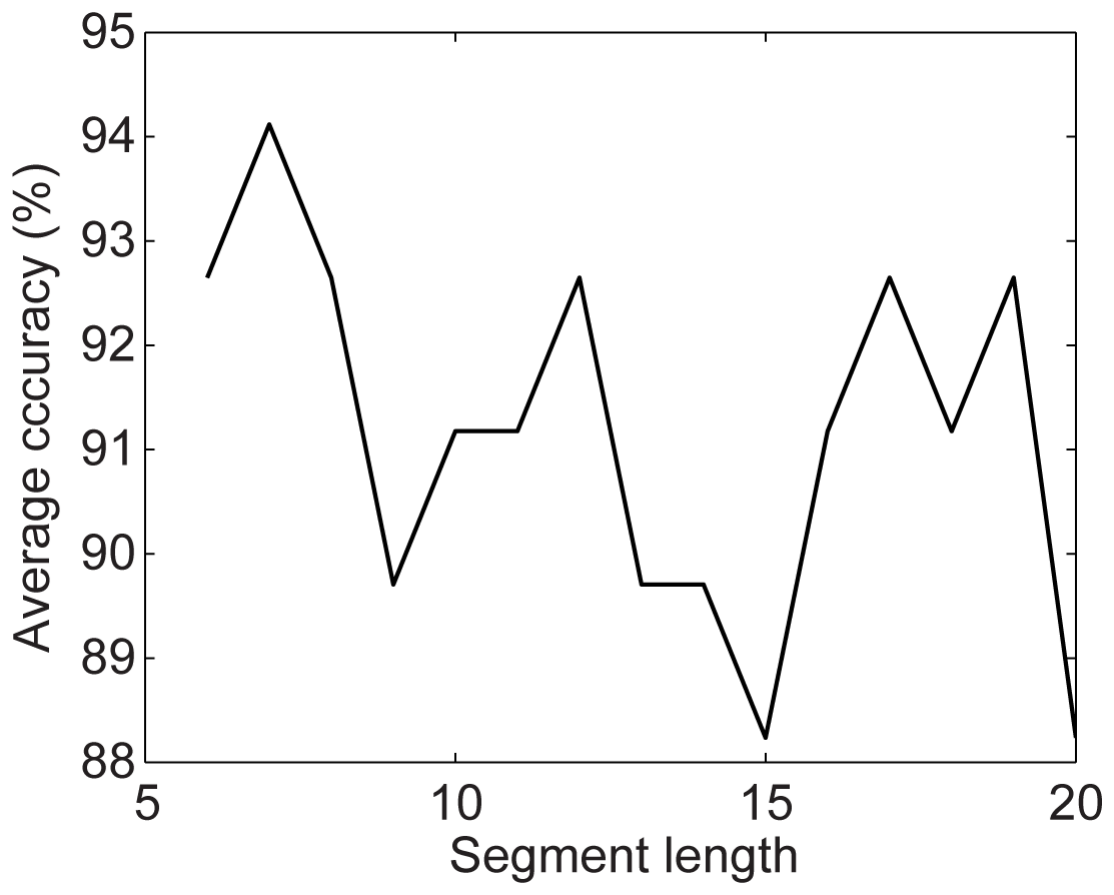
Average accuracy as a function of the number of frames of sub-volumes on the CAD-60 dataset.

**Figure 12:**
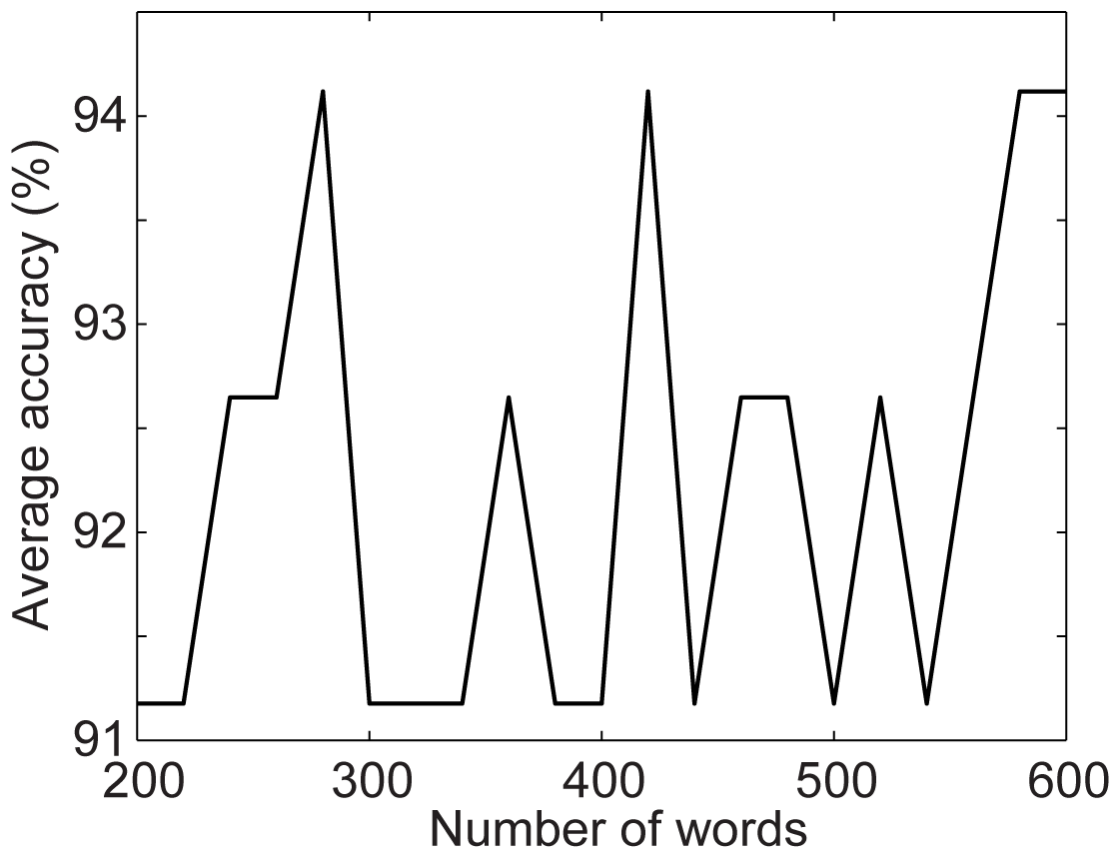
Average accuracy as a function of the number of words in the dictionaries on the CAD-60 dataset.

**Figure 13:**
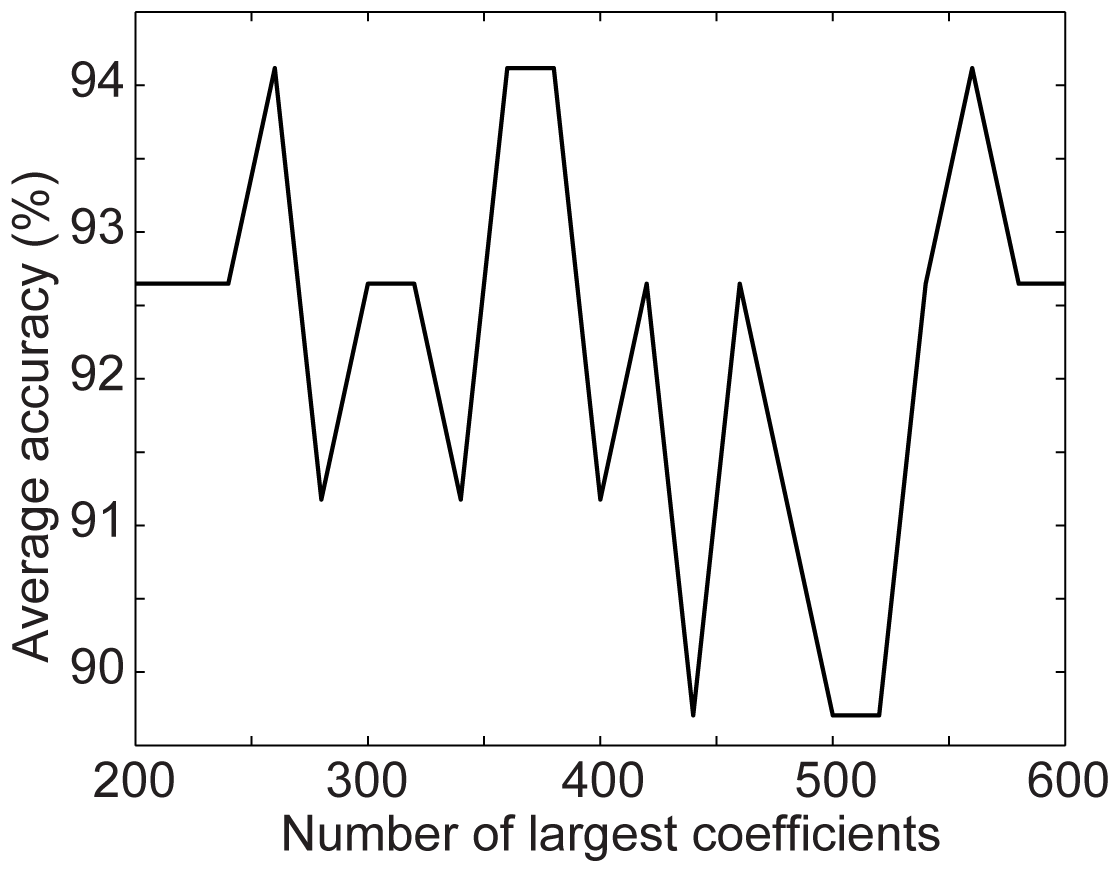
Average accuracy as a function of the number of largest coefficients kept in the sparse histograms on the CAD-60 dataset.

Of the seven parameters, the sampling size 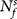, the number of words 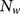, and the number of the largest coefficients 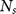 are new in our algorithm while other parameters appeared in other published studies [25], [42]. Therefore, we explore how to choose the values of these three parameters while setting other parameters to the values recommended by other researchers [25], [42]. We run our algorithm with different parameter values on the CAD60 dataset. Fig. 11 shows the average accuracy as a function of the sampling size 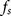 when 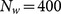 and 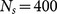; Fig. 12 shows the average accuracy as a function of the number of words 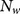 when 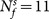 and 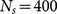; and Fig. 13 shows the average accuracy as a function of the number of the largest coefficients 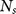 when 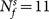 and 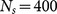. These good results on action recognition obtained under a wide range of parameter settings show that our method is not sensitive to parameter values. Therefore, setting the parameters in our algorithm for good recognition performance is not challenging.

The values of the parameters for all the experiments are listed in Table 7. For simplicity, we set the parameter values the same for the three databases except 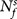, the sampling size along the z-direction, which may depend on the speed of the activities and the frame rate of the videos. As shown in Tables 1, 3, 5, and 6 and Figs. 8–13, there are a range of parameter values in our method that lead to very good performance, which may be further improved by finely tuned parameter values.

**Table 7:** Values of the parameters of our method.

Database	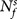	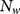	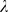	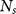	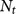	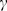	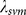
CAD-60	11	400	0.5	400	3	0.01	0.01
MSRAction3D	13	400	0.5	400	3	0.01	0.01
MSRDailyActivity3D	21	400	0.5	400	3	0.01	0.01

The proposed algorithm was implemented in Matlab without any optimization in programming. We evaluated the time performance of our method using Intel(R) Core(TM)2 Duo CPU E8600@3.33 GHz with 64 bit Windows 7 professional SP1 OS. Only one core (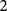 cores available) was used based on single thread programming. We report 4 measures, i.e., the average training time (ATT), the average testing time per video (ATTPV), the average number of training videos (ANTV), the average number of test videos (ANOTV), and the average number of training classes (ANTC) on the three datasets in Table 8.

**Table 8:** Time performance of our method evaluated on the databases CAD-60, MSR Action3D and MSR Daily Activity 3D. **ATT**: Average Training Time (seconds per setting); **ATTPV**: Average Testing Time per Video (seconds per video); **ANTV**: Average Number of Training Videos per Setting; **ANOTV**: Average Number of Testing Videos per Setting; **ANTC**: Average Number of Training Classes per Setting.

Database	ATT	ATTPV	ANTV	ANOTV	ANTC
CAD-60	513.43	**0.50**	51	17	14
MSRAction3D	73.02	**0.03**	114	109	8
MSRDaily Activity3D	125.60	**0.10**	60	60	6

As shown in the table, our method took 0.50, 0.03, and 0.10 seconds/per video to classify the activities of the CAD-60 dataset, the MSR Action3D dataset, and the MSR Daily Activity 3D dataset respectively. The training time was 513.43 seconds, 73.02 seconds, and 125.60 seconds on the CAD-60 dataset, the MSR Action3D dataset, and the MSR Daily Activity 3D dataset respectively. This time performance can be improved significantly by optimized C++ codes running on much faster CPUs. Therefore, our model is a real-time method that can be used in smart environments and deployed in robots for human-robot collaborations.

## Discussion

In this paper we proposed a real-time algorithm that makes use of joint information to recognize human activities. In the first step of the algorithm, videos of 3D movements of body joints are sampled to obtain a set of spatial-temporal 3D volumes, which entail the complex spatial-temporal relationships of joints of human activities at a data size that is much smaller than that of a RGBD volume. Second, RICA is performed on the spatial-temporal 3D volumes to obtain a set of dictionaries of codes that form a sparse representation of human activities. An approximate spare coding scheme is then used to compile a set of spare histograms as features for activity recognition. Finally, a multi-class SVM is used to perform activity recognition. We performed extensive tests on this algorithm on three widely used datasets of human activities. Our results show that this algorithm produces so far the best recognition accuracy on these datasets.

Our algorithm automatically learns discriminative features for activity recognition and is very fast and easy to implement. Since joint information can be obtained by low-cost cameras such as the Microsoft Kinect systems, our algorithm can be used in smart environments and deployed in robots for human-robot collaborations. This model can be improved by the rich information in depth images. To include this information, we will extend the model presented here and our recent model of activity recognition based on multi-scale activity structures [45].

## Supporting Information

Dataset S1 **CAD-60 dataset.**
(RAR)

Dataset S2 **MSR Action 3D dataset.**
(RAR)

Dataset S3 **MSR Daily Activity 3D dataset.**
(RAR)
